# Cloning, Expression, and Immunogenicity of Fimbrial-F17A Subunit Vaccine against* Escherichia coli* Isolated from Bovine Mastitis

**DOI:** 10.1155/2017/3248483

**Published:** 2017-11-29

**Authors:** Wei Chen, Yongxia Liu, Jinhua Yin, Youtian Deng, Tariq Ali, Ju Zhang, Jia Cheng, Sadeeq ur Rahman, Jian Gao, Bo Han

**Affiliations:** ^1^College of Veterinary Medicine, China Agricultural University, Beijing 100193, China; ^2^College of Veterinary Medicine, Shandong Agricultural University, Tai'an 271018, China; ^3^College of Veterinary Sciences and Animal Husbandry, Abdul Wali Khan University, Garden Campus, Mardan, Pakistan

## Abstract

There is a need to identify and select new promising immunodominant antigens that have the ability to provide protective immunity against* E. coli* causing bovine mastitis. Recently we showed that* f17a* was found to be the most prevalent and crucial virulent factor among the pathogenic* E. coli* isolated from bovine mastitis. Here, in this report, the recombinant F17A based subunit vaccine adjuvant with MF59 was tested for immunogenicity against* E. coli* in a murine model. The vaccinated mice did not show any abnormal behavioral changes and histopathological lesions after vaccination. The specific antibody level against F17A was significantly higher in MF59-adjuvant-group, and also lasted for longer duration with a significant (*P* < 0.01) production level of IgG1 and IgG2a. Moreover, we noted higher survival rate in mice injected with F17A-MF59-adjuvant group after challenging with the clinical* E. coli* strain. Our findings of bacterial clearance test revealed that elimination rate from liver, spleen, and kidney in MF59-adjuvant-group was significantly higher than the control group. Finally, the proportion of CD4+T cells was increased, while CD8+ was decreased in MF59-adjuvant group. In conclusion, the current study reveals the capability of F17A-MF59 as a potential vaccine candidate against pathogenic* E. coli* causing mastitis in dairy animals.

## 1. Introduction

The causative agents of coliform bovine mastitis include Gram-negative lactose fermenters including* Escherichia coli*,* Enterobacter* species (*aerogenes* and* cloacae*), and* Klebsiella* [1]. The occurrence of coliform mastitis has been observed at higher level during postparturition period as animals enter their lactation period [2].* E. coli* is commonly recognized as an opportunistic pathogen; however, a few species can lead to serious disease conditions in animals and human. Ruminants have been recognized as asymptomatic carriers of pathogenic and nonpathogenic* E. coli* strains and environmental contamination with ruminants faeces containing* E. coli* is the principal source of dissemination that finally causes bovine mastitis after physical contact [3].

The pathogenic* E. coli* usually produces several virulence factors, which include adhesin, invasins, toxins, capsular, and serum resistance associated factors. Adherence of* E. coli* to host cells is recognized as the first step to colonize the cell surfaces [4]. Thus, these pathogenic* E. coli* carry numerous types of fimbrial and afimbrial adhesins factors including fimbrial adhesins of the P, S, and F17 families and afimibrial adhesins of the AFA family [5] that mediate adherence to the host cells via binding to fibronectin and laminin [6, 7] of the host receptors. Among these factors, F17-fimbriated* E. coli* were found highly prevalent in cattle populations and even thought to be restricted to bovine* E. coli* isolates [8]. The F17 family is comprised of seven variants, F17a, F17b, F17c, F17d, F17e, F17f, and F17g, which are the major subunit protein of the fimbriae encoded by* f17A* gene, and they can be distinguished by specific PCR assays [5, 6]. Furthermore, the F17 fimbriae have been reported to be associated with the pathotypes of* E. coli* [5]. The exact role of the F17 family fimbriae is unknown; however, they are anticipated as target antigens for vaccine development due to their obvious association with virulence and involvement in host-pathogen interaction.

Several new adjuvants have been explored and developed to enhance the immunogenicity of antigens including the popular MF59-adjuvant. MF59 is an oil-in water (o/w) adjuvant consisting of small, uniform, and stable microvesicles prepared from squalene, polysorbate 80 (Tween-80), and sorbitan trioleate (Span-85) [9]. Importantly, this is the only adjuvant licensed for human use since the introduction of alum based adjuvant. Previous studies have confirmed that MF59 possess ideal adjuvant properties such as the favorable safety and strong enhancement of immune responses [9].

Drug resistant* E. coli* recovered generally from food animals and mainly from mastitic cows have often been found to be multidrug resistant [10–12], more likely due to consistent exposure to drugs used for treatment. Therefore, implementation of prophylactic measures such as application of effective vaccine would more likely reduce the chances of occurrence of mastitis, hence exposure to different drugs that are used for treatment against mastitis. Previously, we identified* F17-A* as the most predominant virulent genes found in clinical isolates of* E. coli* recovered from bovine mastitis [13]. Since adherence to the mammary epithelial cells, a prerequisite for colonization during the course of mastitis, is mainly initiated by adhesins such as F17A-, P-, and S-fimbriae [14], which are localized in the outer membrane of* E. coli*, this study was aimed at investigating the immunoprotective effects of recombinant F17A vaccines adjuvant with MF59, as described in this work.

## 2. Materials and Methods

### 2.1. Statement of Ethics

This study was conducted in accordance with the ethical guidelines of China Agricultural University (CAU), Beijing. Furthermore, prior to the initiation of this study, proper ethical approval was granted by the departmental committee of College of Veterinary Medicine, CAU, and all steps possible were taken to avoid animal suffering at any stage of the experiment.

### 2.2. Construction of F17A-MF59-Adjuvant Vaccine

#### 2.2.1. Recombinant F17A Protein

Clinical* E. coli* strain* E-BJ-1* harboring* f17a* gene, previously isolated from bovine mastitis and stored in −80°C, was used in this study. A set of primers based on the* f17a* gene sequence (accession number AF055309.1) [15] was designed P1: 5′-  GGATCC TATGACGGTACAATTAC -3′, containing a* BamHI* site (underlined) and P2: 5′-  CTCGAGTTACTGATAAGCGATGGTG -3′, containing an* Xho I* site (underlined). The PCR conditions were as follows: initial denaturation at 95°C for 5 min, followed by 30 cycles of 95°C for 30 s and annealing at 55°C for 30 s and then 72°C for 60 s, and final extension for 10 min at 72°C.

The resultant PCR amplified* f17a* gene was cloned into the* BamHI*-*XhoI* site of pET28a vector (Promega, Madison, WI, USA) after cleavage with the two restriction endonucleases (Thermo, Waltham, MA, USA). The sequence verified construct was then transformed into* E. coli* BL21 (DE3)-competent cells (TransGen Biotech, Beijing, China).* E. coli* was grown in Luria-Bertani (LB; Difco™, Becton Dickinson, Sparks, MD USA) broth to express F17A protein under the influence of 1 mM IPTG (isopropyl-beta-D-thiogalactopyranoside; Thermo Scientific™) for 4 hours. The cells were then harvested and resuspended in lysis buffer (8 M urea, 50 mM sodium dihydrogen phosphate, 10 mM imidazole, 300 mM sodium chloride, 10 mM Tris, pH 8.0) and lysed by ultrasonication. The recombinant protein was purified and collected with ProteinIso Ni-NTA Resin (TransGen Biotech, Beijing, China) and confirmed by western blot using anti-His monoclonal antibody. The purified protein was processed for renaturation in renaturation solution (0.1 M Tris, 1 mM glutathione, and urea with different concentrations of 1 M, 2 M, 4 M, and 8 M), and Lipopolysaccharide (LPS) was detected and removed as previously described [16]. Then the purified recombinant protein was quantified by the Protein Quantitative Kit (TransGen Biotech, Beijing, China) and adjusted to 600 *μ*g/mL and finally stored at 4°C before use.

#### 2.2.2. F17A-MF59-Adjuvant Vaccine

Each component of MF59 adjuvant, including squalene (4.3%, v/v), Tween-80 (5%, v/v), and Span-85 (5%, v/v), was proportionally dissolved in sodium citrate buffer (10 nM) and mixed homogeneously. The product was emulsified by ultrasonication (2000 W, 3 s/3 s, 5 min) and filtered with a 0.2-*μ*m filter. Then the vaccine was developed by mixing the MF59 emulsion and F17A protein (1 : 1, v/v) and stored at 4°C after vortex shaking for 10 min. In all cases the recombinant F17A protein concentration in the vaccine was maintained at 300 mg/ml.

### 2.3. Animals and Strains

Specific-pathogen-free (SPF) mice (weighing 18 ± 2 g) were purchased from Vital River Laboratory Animal Technology Co. Ltd. (Beijing, China) and housed in a controlled environment (23 ± 2°C, 50 ± 5% humidity, a natural light-dark cycle). All mice were given free access to standard rodent feed (PMI Nutrition International Inc., St. Louis, MO, USA) and tap water. Intraperitoneal (I/P) route was used for inoculation of vaccine as well as for challenging.* E. coli* strain* E-BJ-1* carrying* f17a* gene was firstly confirmed to express F17A by western blot (the primary antibody was from the mice serum vaccinated by F17A-MF59 vaccine for 6 weeks) and then used as the challenging subject in this trial. Cervical dislocation was performed before collection of tissues as previously described [5].

### 2.4. Vaccine Safety Assessment

As elaborated in Table 1, a group of 10 mice was treated with F17A vaccine adjuvant with MF59, and then the clinical features and pathological changes were examined for the next 21 days, whereas ten mice were used as control by inoculating PBS intraperitoneally. Furthermore, complete blood count and blood biochemical profile of vaccinated (*n* = 10) and nonvaccinated (PBS) mice (conducted by China Agricultural University Animal Teaching Hospital) were assayed for comparison to assess adverse outcomes of the formulated vaccine. The following parameters were evaluated: total number of red blood cells (RBC), hemoglobin (HGB), mean corpuscular volume (MCV), red blood cell distribution width (RDW), hematocrit (HCT), mean corpuscular hemoglobin (MCH), mean corpuscular hemoglobin concentration (MCHC), white blood cells (WBC), neutrophils (NE), eosinophils (EO), basophils (BA), lymphocytes (LY), monocytes (MO), platelet count (PLT), mean platelet volume (MPV), alkaline phosphatase (ALP), alanine aminotransferase (ALT), aspartate aminotransferase (AST), inorganic phosphate (IP), serum calcium (Ca), glucose (GLU), blood urea nitrogen (BUN), creatinine (CR), total bilirubin (TBIL), total cholesterol (TCHO), albumin (ALB), and triglyceride (TG). Student's* t*-test was used for the comparison of blood parameters between vaccinated and nonvaccinated group. A *P* value less than 0.05 was considered significant.

### 2.5. Immunogenicity Assay

To evaluate the immunological characteristics of vaccinated mice, a total of 30 mice were randomly divided into three groups with ten mice in each group, namely, group C, group F, and group V, which represented the control group treated with PBS, F17A nonadjuvant group, and F17A-MF59-adjuvant group, respectively (Table 1). During the next eight weeks, antibody against F17A vaccine was detected by ELISA every two weeks, and at 6th week of postinoculation the antibody subtyping (IgG1, IgG2a, IgA, and IgM) was carried out by the Rapid Antibody Isotyping Kit (Zymed Laboratories Inc., South San Francisco, CA, USA) according to the instructions of manufacturer.

### 2.6. Challenging and Survival Trials

As shown in Table 1, twenty mice were randomly divided into two groups. Group C that was comprised of ten mice was inoculated with plain PBS and group V was injected with F17A-MF59-adjuvant vaccine and then after six weeks these mice were challenged by clinical* E. coli* with a dose of 4 × 10^8^ CFU. The comparative survival rate in both groups was observed for 21 days after challenging.

### 2.7. Bacterial Load Assay

In this part of experiment, two groups were injected with plain PBS and MF59-adjuvant vaccine, respectively, and then each group was challenged by clinical* E. coli E-BJ-1* strain with 8 × 10^7^ CFU per mouse six weeks after inoculation (Table 1). Five mice from each group were fasted overnight and euthanized, respectively, 1, 3, 5, and 7 days after challenging. Then, the liver, spleen, and kidney were resected under aseptic conditions and weighed. The bacterial load in each organ was detected by colony counting method.

### 2.8. Histopathological Analysis

As shown in Table 1, after nine days of challenging, the mice were operated on. The liver, spleen, and kidney were incised and cut into ~2 cm^3^ pieces and then fixed in 10% buffered formalin. The fixed organs were dehydrated in a graded series of ethanol, cleared in xylene, and embedded in paraffin. Then the histological slices (4 *μ*m) were stained with hematoxylin and eosin and evaluated under optical-microscope (Olympus, Japan).

### 2.9. Classification of T Lymphocytes

Seventy-two hours after challenging, blood samples of the mice were collected into the anticoagulant tube by posterior orbital venous plexus. According to the kit instruction, the samples were divided into 200 *μ*L in each tube and stained by PEcy5-anti-CD3, FITC-anti-CD4, and PE-anti-CD8 (eBioscience, San Diego, CA, USA) monoclonal antibodies for 20 min in the absence of light. Then the hemolysin was added and incubated for 10 min at 4°C. After centrifugation and washing with PBS, the cells were resuspended in 1% paraformaldehyde and then detected by FACS Calibur Flow Cytometry (New Jersey, USA).

### 2.10. Statistical Analysis

All analyzed parameters were expressed as mean ± standard deviation (SD). Statistical analysis was performed using Chi-square test by SPSS version 19.0 (SPSS Inc., Chicago, IL, USA). Kaplan–Meier survival curves were analyzed using the log rank test. Statistical significance was defined as *P* < 0.05.

## 3. Results

### 3.1. Cloning and Expression of F17A Antigen

**Figure 1:**
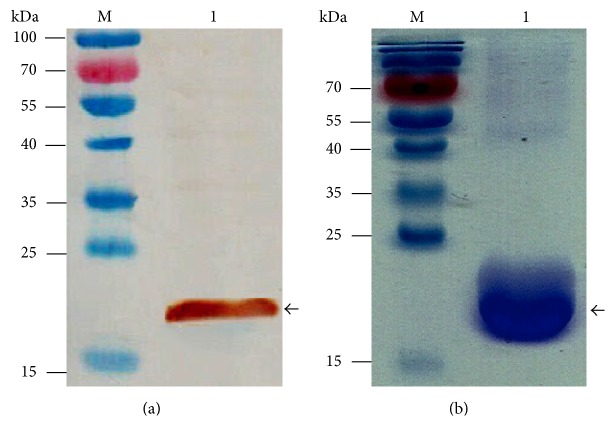
*Expression and confirmation of F17A protein*. (a) F17A protein was produced under the influence of IPTG in expression host* E. coli* BL21 (DE3). The expressed protein was Ni-NTA purified and run on gradient SDS-PAGE gel and immunoblotted against anti-His antibody. A dominant band of ~20 kDa of the expected size is indicated as recombinant F17A protein. (b) F17A protein run on SDS-PAGE and stained indicated by the expected size of ~20 kDa. Line M: protein marker; line 1: F17A protein.

We PCR amplified* f17a* from* E. coli* isolated from bovine mastitis as previously reported from our lab [13]. The amplified PCR product was digested with* BamHI* and* XhoI* and cloned into pET28a vector that was already restricted with the same enzymes. The sequence verified construct (GenBank Accession Number AF055309.1) was then transformed into* E. coli* BL21 (DE3) and expressed under the influence of IPTG. The resultant recombinant protein was run on SDS-PAGE gel that revealed successful expression of the protein indicated by the expected size (Figure 1(b)) and was confirmed by western blotting using anti-his antibody (Figure 1(a)). For vaccine preparation, we produce a higher amount of protein that we then purified by Ni-NTA resin. Our analysis of the LPS levels in the purified protein indicated a level of less than 0.3 EU/*μ*g.

### 3.2. Development of F17A-MF59-Adjuvant Vaccine

We prepared F17A-MF59-adjuvant vaccine by mixing F17A protein and MF59 adjuvant. The final concentration of the recombinant protein F17A was maintained at 300 mg/ml. The vaccine was found homogeneous and its composition and pH remained unchanged for twelve months at 4°C.

### 3.3. Safety Assessment

We administered a double dosage of F17A*-*MF59 vaccine to mice in order to evaluate the safety level. Our results showed that mice survived the following 21 days without any clinical signs. Furthermore, no significant differences were observed in complete blood count and blood biochemical profiles between vaccinated and control group (Supplementary Tables 1 and 2). Likewise, no pathological changes were observed in vaccinated animals upon postmortem examination (not shown), which suggest that the tested vaccine exhibited no obvious toxicity during the tested period even when injected at double dose rate.

### 3.4. Immunogenicity

**Figure 2:**
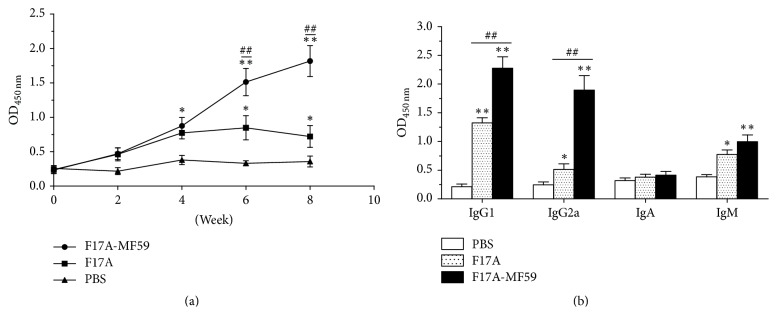
*Immunological study of mice vaccinated with F17A and F17A-MF59-adjuvant vaccines*. (a) Antibody level in immunized mice serum; (b) antibody subtypes in immunized mice serum. ^*∗*^*P* < 0.05, ^*∗∗*^*P* < 0.01, as compared to PBS group; ^##^*P* < 0.01, as compared to F17A group.

Our results showed that the antibody level against F17A-MF59-adjuvant vaccine was significantly higher than the nonadjuvant vaccine. As depicted from Figure 2(a), the antibody level of MF59-adjuvant group continued to rise and increased to a considerable higher level at 8th week (*P* < 0.01). However, in comparison to this, the antibody titer of the nonadjuvant group was maintained at lower level and also declined significantly (*P* < 0.05) at 6th week after vaccination.

Next, our analysis of the antibody isotyping of the serum samples obtained from mice at 6th week of postinoculation indicated that that IgG1 was predominantly found in both F17A-MF59-adjuvant and F17A nonadjuvant groups suggesting that humoral immunity mainly occurred in response to F17A. Importantly, the IgG1 level in MF59-adjuvant group was found significantly (*P* < 0.01) higher than the nonadjuvant F17A group. Furthermore, IgG2a production against F17A-MF59 group was also found significantly higher than the nonadjuvant group (*P* < 0.01), which indicates that MF59-adjuvant can also effectively promote cellular immunity mediated by Type-I helper cells (Th1) response. Finally, the level of IgM in mice vaccinated with F17A-MF59 vaccine (*P* < 0.01) and F17A protein (*P* < 0.05) was found obviously higher than the mice treated with PBS (Figure 2(b)).

### 3.5. Survival Test

**Figure 3:**
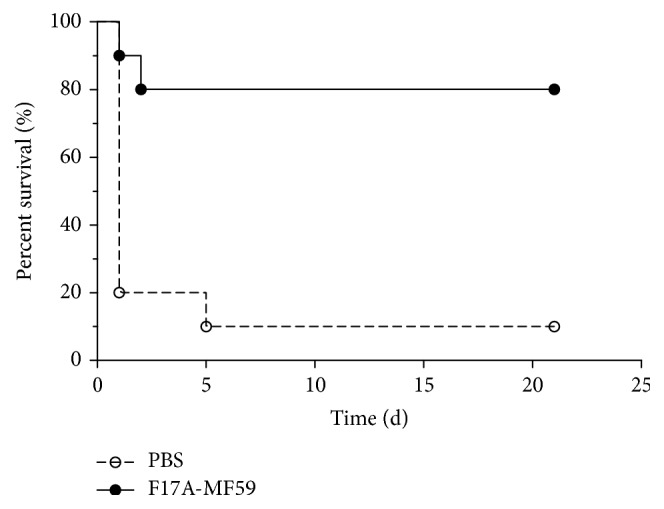
Percent survival rate of mice after being challenged with* E. coli* and vaccinated with F17A-MF59 vaccine.

After six weeks of vaccination, we challenged the vaccinated mice (*n* = 10) with* E. coli* strain* E-BJ-1* at a dose rate of 4 × 10^8^ CFU and mice were observed. As a control, ten nonvaccinated mice were also infected. After inoculation, mice presented clinical features such as depression, anorexia, and fatigue. We observed high mortality rate at 1st day in control nonvaccinated group leaving a single mouse surviving at 21st day revealing 90% mortality, whereas, in the group vaccinated with F17A-MF59-adjuvant vaccine, only two mice died during the first two days, and then the mice gradually recovered to normal conditions and eight mice survived until the 21st day, representing a total mortality of 20% (Figure 3).

### 3.6. Bacterial Load Assessment

**Figure 4:**
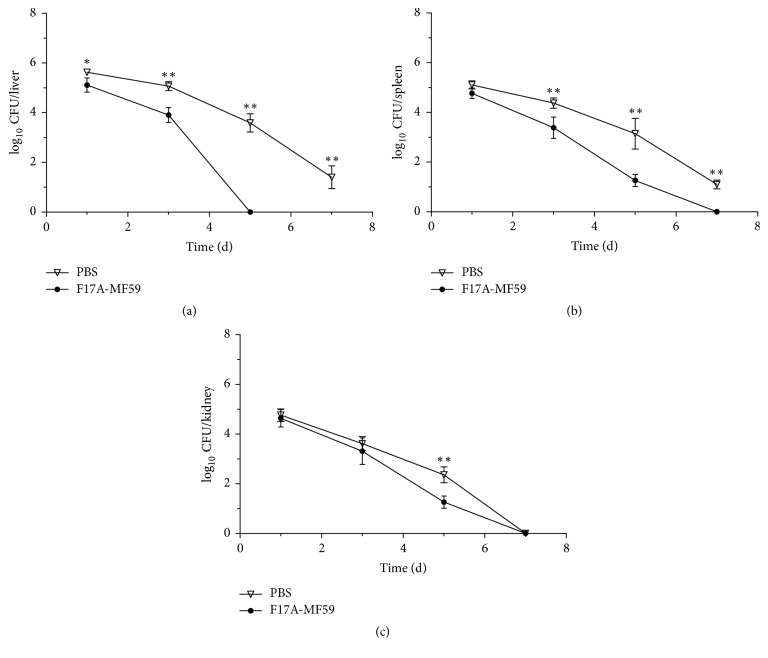
*Dynamic curve of bacterial load in liver (a), spleen (b), and kidney (c)*. ^*∗*^*P* < 0.05, ^*∗∗*^*P* < 0.01, as compared to F17A-MF59 group.

Our analysis of the bacterial load assessment of the challenged mice infected with* E. coli *at a dose rate of 8 × 10^7^ CFU showed that, in the liver, the bacterial load in F17A-MF59 group decreased with a greater degree in comparison to PBS group at 1st day (*P* < 0.05), and this decrease became more significant in the following days, and finally bacteria were almost completely eliminated at 5th day in F17A-MF59-adjuvant group (*P* < 0.01). A similar trend was also observed in the spleen, that is, the bacterial load in F17A-MF59 group was found significantly lower (*P* < 0.01) than the PBS group on 3rd day after challenge and bacterial load became nearly zero at 7th day (*P* < 0.01) in F17A-MF59 group. We also observed similar findings in kidney at 5th day (*P* < 0.01) as elaborated in Figure 4.

### 3.7. Histopathological Examination

**Figure 5:**
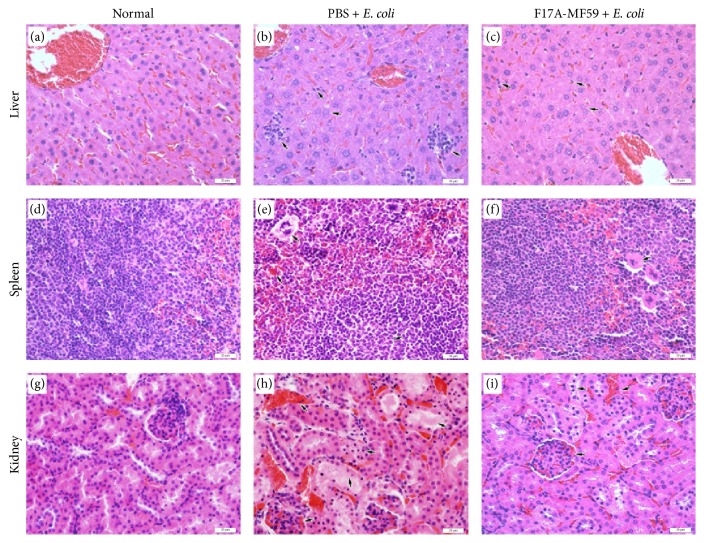
*Histological analysis of mice liver, spleen, and kidney*. Hematoxylin-eosin staining. The scale indicated as 20 *μ*m. (a) to (c) were histological sections of mice liver, (d) to (f) were mice spleen, and (g) to (i) were mice kidney. (a), (d), and (g) were the tissues from control group; (b), (e), and (h) were the tissues from PBS +* E. coli* group; (c), (f), and (i) were from F17A-MF59 +* E. coli* group. (b) Hepatocyte degeneration with cellular swelling and dissolution and disappearance of nucleus; structure disturbance of hepatic cords; Kupffer cells increased and lymphocyte aggregation (arrows) occurred. (c) Swelling of hepatocytes and dissolution and disappearance of nucleus; structural abnormality of hepatic cords; Kupffer cells (arrows) increased. (e) Serious hyperaemia and megakaryocytes (arrows) increased in splenic red pulps. (f) Megakaryocytes (arrows) increased in splenic red pulps. (h) Hyperaemia in the glomeruli capillaries, congestion (arrows) in renal tubular interstitium, degeneration of renal tubular epithelial cells presenting swelling, karyopyknosis, and karyolysis, and reddish material (arrows) exuded in lumen renal tubulars. (i) Mild hyperaemia in the glomeruli capillaries, mild congestion (arrows) in renal tubular interstitium, and local karyopyknosis (arrows) of renal tubular epithelial cells.

The histopathological changes in liver, spleen, and kidney in the mice challenged with* E. coli* strain* E-BJ-1* are shown in Figure 5. Our results revealed increased production of Kupffer cells, lymphocytes infiltration, lymphocytic aggregation at hepatic lobule, disintegration of hepatic cords structure, hepatocytes swelling, and degeneration in the group of mice injected with PBS (Figure 5(b)), while F17A-MF59 group depicted similar pathological changes, albeit to a lesser degree as compared to the PBS group (Figure 5(c)). Moreover, in the spleen of PBS group, germinal center in white pulp was not clear, the cells were diminished, red pulp giant cells increased in the blood sinus, and obvious hyperemia was also observed (Figure 5(e)). However, only the giant cells were increased in the blood sinus of red pulp in the spleen of F17A-MF59 group (Figure 5(f)). The histopathological examination of kidney showed serious congestion in the renal tubular interstitium in the PBS group, as well as hyperaemia in the glomeruli capillaries, swelling, and degeneration of renal tubular epithelial cells, and karyopyknosis of few epithelial cells (Figure 5(h)), while F17A-MF59 group showed slight congestion in the renal tubular interstitium, along with hyperaemia in the glomeruli capillaries and also less extent of karyopyknosis and degeneration in epithelial cells (Figure 5(i)).

### 3.8. Classification of T Lymphocytes

**Figure 6:**
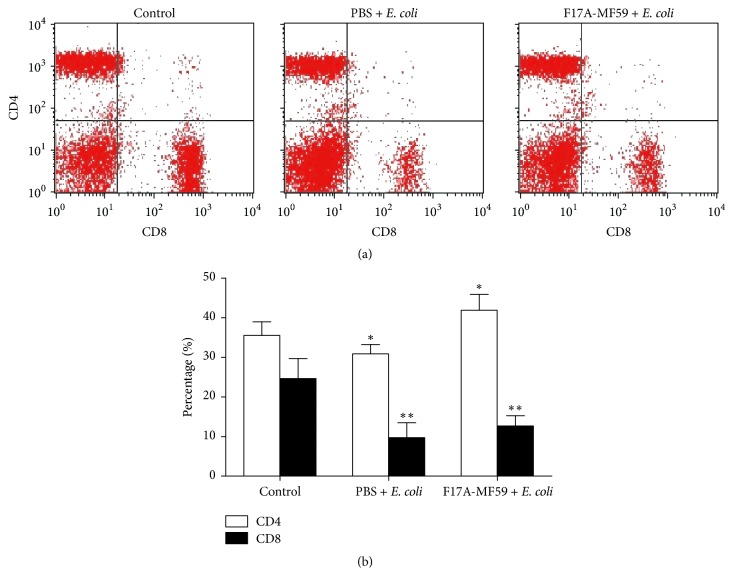
*Alternation of CD4+∖CD8+ T lymphocytes in mice caused by F17A-MF59-adjuvant vaccine as compared to control*. ^*∗*^*P* < 0.05, ^*∗∗*^*P* < 0.01.

We assessed T lymphocytes subsets in the peripheral blood of mice in order to determine the effect of F17A-MF59-adjuvant vaccine on the immune function (Figure 6). Our results showed that the frequency of CD4+ (*P* < 0.05) and CD8+ T cells (*P* < 0.01) in PBS group was all significantly decreased after challenging with the clinical* E. coli *strain, whereas in the F17A-MF59 group, only CD8+ T cells population was decreased, but with a slower pace than in the PBS group (*P* < 0.01), while the CD4+ T cells were significantly increased (*P* < 0.05).

## 4. Discussion

*E. coli* strain is an important causative agent of bovine mastitis in dairy cows. The pathogenicity and duration of the disease vary greatly, and mostly they depend upon the individual response of the cow. Currently, the coli mastitis vaccines administered during early months of lactation have shown a promising effect to some extent against the coliform bovine mastitis [17, 18].* E. coli* harboring* f17* gene, which encodes for F17 protein of fimbriae and is the most prevalent virulence factor, are responsible for several important diseases in cattle and other animals, including calf diarrhea and bovine mastitis [4]. This study was designed with the aim of evaluating the comparative immunological characteristics of MF59-adjuvant and nonadjuvant F17A vaccine.

Fimbriae are filamentous appendices, naturally localized on the bacterial surface, and are commonly responsible for mediating adhesion to the host cells and can promote specific binding to the receptors on surface of host cells [19]. Adherence to the host cells establishes the initial occurrence of* E. coli* pathogenesis. Several recent studies reported F17 as the most prevalent fimbriae of bovine* E. coli* isolates and F17A as the main subunit of F17 protein [4, 13]. Secondly, F17A is localized in the outer membrane of* E. coli* that could potentially be exposed. Thus, based on the abundance of F17A in our clinical isolates and its localization in the outer membrane, we chose to test its immunogenicity potential. Outer membrane or secreted proteins that have been previously shown as crucial virulent factors in bacterial pathogenesis have been proven as promising vaccinal candidates when alone or in combination with other parts that have been tested [20–24]. F17A related adhesins (formerly known as FY) have been shown to promote inhabitable* N*-acetyl-D-glucosamine (GlcNac) hemagglutination of bovine erythrocytes and* in vitro* adhesion to the brush border of intestinal calf villi [25]. We anticipate that the immunological protection was ensured by the antibodies directed against F17A pili. It has been previously shown that newborn calves of F17 antigen vaccinated cows were found to protect against enterotoxigenic* E. coli* and that was attributed to the level of antibodies against F17 fimbriae in the colostrum of vaccinated cows [26].

Although the adjuvant vaccines have been used for more than 70 years, the specific mechanism of adjuvant is still unclear. For example, aluminum salt, the major adjuvant, was assumed to display the depot effect by absorbing the antigen and keeping it for longer duration at the site of injection, thereby maintaining a comparably high concentration for phagocytes uptake. But this mechanism cannot be considered for all the immunological traits mediated by alum and other vaccine adjuvants [27, 28]. Similarly, little is known about the exact mechanism of MF59 adjuvant; however, it has been detected in muscles and T-cell areas within lymph nodes [29]. Furthermore, injection of MF59 adjuvant can also result in an obvious aggregation of macrophages at the site of injection, while this phenomenon was significantly suppressed in mice deficient in chemokines receptor 2, suggesting that one of the effects of MF59 may be to activate the cells resident at the injection site to produce chemokines [30]. Additionally, several studies showed that MF59 can create a local immune-stimulating environment and directly trigger human immune cells like T cells* in vivo* [31].

Our reported subunit MF59 adjuvant vaccine did not reveal any treatment-associated safety issues. Only local inflammatory responses at the site of injection were reported through histopathology, which suggest that this adjuvant just possess a low order of local reactogenicity and not any potential for systemic toxicity. Several studies reported that MF59 have no adverse effect in MF59-adjuvant vaccine group through comparison of vaccine group and MF59 alone [9]. In line with our findings, previous studies found that the antigen combined with MF59 adjuvant generated higher specific antibody titers than the nonadjuvant antigens [32]. In addition, data regarding the clinical immunogenicity showed that MF59-adjuvant vaccine elicits a strong antibody response as reported by others [33].

In the present study, as compared to the non-MF59-adjuvant group, mice in F17A-MF59-adjuvant group presented higher antibody level against F17A with significantly higher production of IgG1 and IgG2a indicating that not only is this vaccine eliciting humoral immunity, but also it effectively promoted cellular immune response. Although IgM was not the dominating antibody subtype against F17A in the current study, its level in mice vaccinated with F17A-MF59 vaccine was still higher than that treated with F17A alone. The humoral immune response elicited by most pathogens is commonly characterized by an early rise of antigen-specific immunoglobulin (IgM), followed by affinity maturation, isotype switching, and the ensuing rise in antigen-specific IgG, IgA, and IgE antibodies. In the early infection, IgM is first class of antibodies generated during primary antibody response but for shorter lifespan; then it is bound with the invading pathogen and they aggregate together to be phagocytized by phagocytes [34].

In the current study, the results of bacterial clearance test and histopathology revealed that the mice in F17A-MF59-adjuvant group showed greater efficiency to eliminate the clinical* E. coli* than the control group, in terms of time and bacterial load, especially in liver and spleen, as well as presenting slight degree of histopathological lesions. Regardless of its mechanism of action, the obvious effect of using MF59-adjuvant was that it enhanced the functional and protective antibody responses and/or induced strong T-cell response to several different types of antigens [35]. CD4+ T cells, which are also known as helper T-cell, can release a variety of lymphokines in the immune response and type IV allergy, resulting in inflammatory response and clearing the antigen of interest; CD8+ T cells, also known as cytotoxic T-cell, can kill the target cells infected by pathogen via the release of perforin and other factors in the immune effect phase [36].

Finally, the effect of F17A-MF59 vaccine on immunological function of the treated mice and its response on T lymphocytes subsets in peripheral blood were evaluated in the current study. As compared with the normal mice, percentages of CD4+ and CD8+ T cells were all significantly decreased in PBS +* E. coli* group, whereas in F17A-MF59 +* E. coli* group, percentage of CD4+ T cells percent increased, while CD8+ T cells percent became lower but with a less extent than PBS +* E. coli* group. This may indicate that, after challenge, the immune function of the mice was impaired by* E. coli* infection, but still MF59 could obviously enhance the humoral immune response; meanwhile, the cellular immune response was suppressed, but the degree was still lower than that of PBS group; this may be due to the fact that* E. coli* is an extracellular pathogen.

## 5. Conclusion

Our previous findings show that* f17a *is a crucial virulent factor of* E. coli*—isolated from bovine mastitis—and coherently this work inferring F17A to elicit protective immune response in a murine model of infection suggests that F17A is a promising vaccine candidate that could potentially be combined with other immunogens to develop an effective recombinant vaccine against bovine mastitis.

## Figures and Tables

**Table 1:** Treatment in animal experiments.

Experiment	Groups	Treatment		Time
		Inoculum	*E. coli* challenge	
Safety assay	C: *n* = 10 V: *n* = 10	400 *μ*L/mouse	None	21 days

Immunogenicity	C: *n* = 10 F: *n* = 10 V: *n* = 10	200 *μ*L/mouse	None	8 weeks

Survival test	C: *n* = 10 V: *n* = 10	200 *μ*L/mouse	4 × 10^8^ CFU, 6 weeks after inoculation	21 days

Bacteria clearance test	C: *n* = 20 V: *n* = 20	200 *μ*L/mouse	8 × 10^7^ CFU, 6 weeks after inoculation	7 days

Histopathology	C: *n* = 5 V: *n* = 5	200 *μ*L/mouse	8 × 10^7^ CFU, 6 weeks after inoculation	9 days

Classification of T lymphocytes	C: *n* = 5 V: *n* = 5	200 *μ*L/mouse	8 × 10^7^ CFU, 6 weeks after inoculation	72 hours

C: control group treated with PBS; V: vaccinated group; F: treated only with F17A.

## Supplementary Materials

Supplementary Materials Supplementary Table  1. Complete blood count (*n* = 10) of vaccinated (experimental) and nonvaccinated mice (control). Supplementary Table  2. Complete blood count (*n* = 10)  of vaccinated (experimental) and nonvaccinated mice (control).
