# Supplementary material for: Cloning, Expression, and Immunogenicity of Fimbrial-F17A Subunit Vaccine against Escherichia coli Isolated from Bovine Mastitis
Source: Biomed Res Int. 2017 Nov 29;2017:3248483. doi: 10.1155/2017/3248483 (PMC5733191; doi:10.1155/2017/3248483)
Supplement: Supplementary Materials — Supplementary Table 1. Complete blood count (n = 10) of vaccinated (experimental) and nonvaccinated mice (control). Supplementary Table 2. Complete blood count (n = 10) of vaccinated (experimental) and nonvaccinated mice (control). [file 3248483.f1.docx]

**Supplementary documents**

Complete blood count and blood biochemical profile of vaccinated (n=10) and non-vaccinated (PBS) mice was assayed for comparison in order to assess the toxicity or adverse effects of the formulated vaccine. Student-t test was used for the comparison of blood parameters between vaccinated and non-vaccinated group. A *P* value less than 0.05 was considered significant.

Supp. Table-1 Complete blood count (n=10) of vaccinated (experimental) and non-vaccinated mice (control)

| Item | Control group | Experimental group | *P* value |
| --- | --- | --- | --- |
| RBC | 10.5 ± 0.37 (×10^6^/μL) | 10.8 ± 0.81 (×10^6^/μL) | > 0.05 |
| HGB | 132.27 ± 5.48 (g/L) | 136.41 ± 7.29 (g/L) | > 0.05 |
| MCV | 41.2 ± 2.81 (fL) | 42.3 ± 2.23 (fL) | > 0.05 |
| RDW | 17.83 ± 2.8 (CV/fL) | 16.92 ± 3.4 (CV/fL) | > 0.05 |
| HCT | 42.7 ± 2.04 (%) | 44.1 ± 2.79 (%) | > 0.05 |
| MCH | 13.1 ± 0.54 (pg) | 13.6 ± 0.47 (pg) | > 0.05 |
| MCHC | 33.29 ± 1.56 (g/L) | 34.02 ± 2.06 (g/L) | > 0.05 |
| WBC | 8.03 ± 1.27 (×10^3^/μL) | 9.14 ± 2.15 (×10^3^/μL) | > 0.05 |
| NE | 0.19 ± 0.04 (×10^3^/μL) | 0.20 ± 0.05 (×10^3^/μL) | > 0.05 |
| EO | 0.004 ± 0.002 (×10^3^/μL) | 0.004 ± 0.001 (×10^3^/μL) | > 0.05 |
| BA | 0.22 ± 0.09 (×10^3^/μL) | 0.27 ± 0.12 (×10^3^/μL) | > 0.05 |
| LY | 1.72 ± 0.14 (×10^3^/μL) | 1.87 ± 0.18 (×10^3^/μL) | > 0.05 |
| MO | 0.22 ± 0.04 (×10^3^/μL) | 0.23 ± 0.07 (×10^3^/μL) | > 0.05 |
| PLT | 782.3 ± 167.2 (×10^3^/μL) | 754.9 ± 191.3 (×10^3^/μL) | > 0.05 |
| MPV | 5.82 ± 1.83 (fL) | 5.71 ± 1.75 (fL) | > 0.05 |

Supp. Table-2 Complete blood count (n=10) of vaccinated (experimental) and non-vaccinated mice (control)

| Item | Control group | Experimental group | *P* value |
| --- | --- | --- | --- |
| ALP | 107.4 ± 15.8 (U/L) | 99.8 ± 19.7 (U/L) | > 0.05 |
| ALT | 17.1 ± 5.2 (U/L) | 19.3 ± 6.9 (U/L) | > 0.05 |
| AST | 103.42 ± 17.85 (U/L) | 92.74 ± 21.32 (U/L) | > 0.05 |
| P | 2.41 ± 0.31 (mmol/L) | 2.78 ± 0.27 (mmol/L) | > 0.05 |
| Ca | 2.27 ± 0.52 (mmol/L) | 2.17 ± 0.42 (mmol/L) | > 0.05 |
| GLU | 8.23 ± 1.86 (mmol/L) | 8.92 ± 2.73 (mmol/L) | > 0.05 |
| BUN | 7.24 ± 0.91 (mmol/L) | 6.87 ± 1.02 (mmol/L) | > 0.05 |
| Cr | 51.17 ± 6.05 (μmol/L) | 48.26 ± 5.17 (μmol/L) | > 0.05 |
| TBIL | 0.87 ± 0.16 (μmol/L) | 0.84 ± 0.08 (μmol/L) | > 0.05 |
| TCHO | 2.38 ± 0.51 (mmol/L) | 2.44 ± 0.62 (mmol/L) | > 0.05 |
| ALB | 28.37 ± 2.14 (g/L) | 29.08 ± 1.42 (g/L) | > 0.05 |
| TG | 1.25 ± 0.37 (mmol/L) | 1.31 ± 0.44 (mmol/L) | > 0.05 |

_Total number of red blood cells (RBC), hemoglobin (HGB), mean corpuscular volume (MCV), red blood cell distribution width (RDW), hematocrit (HCT), mean corpusular hemoglobin (MCH),  mean corpusular hemoglobin concentration (MCHC), white blood cells (WBC), neutrophils (NE), eosinophils (EO), basophils (BA), lymphocytes (LY), monocytes (MO), platelet count (PLT), mean platelet volume (MPV), alkaline phosphatase (ALP), alanine aminotransferase (ALT), aspartate aminotransferase (AST),  inorganic phosphate (IP), serum calcium (Ca), glucose (GLU), blood urea nitrogen (BUN), creatinine (CR), total bilirubin (TBIL), total cholesterol (TCHO), albumin (ALB),  triglyceride (TG)._
